# Protective Effects of N-Acetylcysteine Against Acrylamide-Induced Lung Toxicity via Regulation of GSK-3β/Nrf2/NF-κB Signaling: Molecular and Immunohistochemical Evidence

**DOI:** 10.3390/toxics14060492

**Published:** 2026-06-04

**Authors:** Amira Osman, Medhat Taha, Sara Abubakr, Nermeen H. Lashine, Rasha Abd Elrahman, Ahmed Mohsen Faheem, Noha M. Halloull, Omnia Hassan Megahed, Nehal E. Refaay, Azza I. Farag, Rania G. Elkatary, Eman Mohamad El Nashar, Mohammed E. Elmitwalli, Hend Ibrahim Abd Elhalim, Kareem Gomaa Al Sayed Ali, Eman Mahmoud FaragAllah, Noha Hammad Sakr

**Affiliations:** 1 Department of Histology and Cell Biology, Faculty of Medicine, Kafrelsheikh University, Kafr El Sheikh 33516, Egypt; mero.osman@med.kfs.edu.eg; 2 Department of Anatomy and Histology, Faculty of Medicine, Mutah University, Karak 61710, Jordan; 3 Department of Human Anatomy and Embryology, Faculty of Medicine, Mansoura University, Mansoura 35516, Egypt; sara_abubakr@mans.edu.eg (S.A.); nermeenlashine@mans.edu.eg (N.H.L.); 4 Department of Anatomy, Al-Qunfudah Medical College, Umm Al-Qura University, Al-Qunfudhah 28814, Saudi Arabia; 5 Faculty of Medicine, Damietta University, Damietta 34517, Egypt; rashaeissa123@du.edu.eg; 6 Department of Medical Biochemistry and Molecular Biology, Faculty of Medicine, Mansoura University, Mansoura 35516, Egypt; dr_ahmed1986@yahoo.com; 7 Forensic Medicine and Clinical Toxicology, Faculty of Medicine, Zagazig University, Zagazig 44519, Egypt; nohahalol1981@gmail.com (N.M.H.); dr_omnia2011@hotmail.com (O.H.M.); 8 Department of Physical Therapy, College of Applied Medical Sciences, Qassim University, Buraidah 51452, Saudi Arabia; n.refaei@qu.edu.sa (N.E.R.); az.ali@qu.edu.sa (A.I.F.); 9 Clinical Pharmacology Department, Faculty of Medicine, Mansoura University, Mansoura 35516, Egypt; rania_al_qatary@mans.edu.eg; 10 Department of Anatomy, College of Medicine, King Khalid University, Abha 62529, Saudi Arabia; enshar@kku.edu.sa; 11 Histology Department, Faculty of Medicine, Al-Azhar University, New Damietta 34517, Egypt; d.mohamed.elmowafe20@gmail.com; 12 Histology and Cell Biology Department, Faculty of Medicine, Tanta University, Tanta 31527, Egypt; hend.abdelhaleem@med.tanta.edu.eg; 13 Department of Pathology, Faculty of Medicine New Damietta, Al Azhar University, New Damietta 34517, Egypt; kareempatho2022@gmail.com; 14 Physiology Department, Faculty of Medicine, Zagazig University, Zagazig 44519, Egypt; noorhema4@gmail.com; 15 Human Anatomy and Embryology, Faculty of Medicine, Kafr Elsheikh University, Kafrelsheikh 33516, Egypt; nohasakr851@gmail.com

**Keywords:** acrylamide, N-acetylcysteine, lung injury, oxidative stress, inflammation, Nrf2, NF-κB

## Abstract

Background: Acrylamide (ACR), a toxic compound formed during high-temperature cooking of carbohydrate-rich foods, is known to induce multi-organ toxicity, including oxidative and inflammatory lung injury. N-Acetylcysteine (NAC), a precursor of glutathione (GSH), possesses potent antioxidant and anti-inflammatory properties that may counteract ACR-induced pulmonary damage. This study investigated the protective effects of NAC against ACR-mediated lung toxicity, with an emphasis on the GSK-3β/Nrf2/NF-κB signaling axis. Methods: Forty male Wistar rats were allocated into four groups: control, NAC (250 mg/kg/day), ACR (50 mg/kg/day), and NAC + ACR. After 11 days of treatment, lung tissues were examined histopathologically using H&E, PAS, and Masson’s trichrome stains. Oxidative stress biomarkers (MDA, SOD, GPx, CAT, GSH) were quantified biochemically. Immunohistochemistry and qRT PCR assessed expression of Nrf2, NF-κB, IL-1β, and Caspase 3, while ELISA measured TNF α, IL-6, Bax, Bcl 2, and GSK 3β. Results: ACR exposure resulted in severe lung injury characterized by alveolar wall edema, epithelial hyperplasia, leukocytic infiltration, goblet cell hyperplasia, and peribronchiolar collagen deposition. These pathological changes were accompanied by a marked increase in MDA, NF-κB, IL-1β, TNF α, IL-6, Bax, Caspase 3, and GSK 3β, together with significant reductions in antioxidant enzymes and Nrf2/HO 1/NQO1 expression. NAC co-administration significantly ameliorated ACR-induced lung damage, restoring normal histological architecture, reducing fibrosis, and normalizing goblet cell activity. NAC also reversed oxidative stress, enhanced Nrf2 and downstream antioxidant responses, suppressed NF-κB-mediated inflammation, and mitigated apoptosis. Notably, NAC downregulated ACR-induced GSK 3β activation, thereby contributing to balanced redox and inflammatory signaling. Conclusions: NAC confers significant protection against ACR-induced pulmonary toxicity through its antioxidant, anti-inflammatory, and anti-apoptotic activities. These effects are mediated, at least in part, by modulation of the GSK 3β/Nrf2/NF-κB pathway. NAC demonstrates promising therapeutic potential for preventing chemically induced lung injury.

## 1. Introduction

Acrylamide (ACR), a toxic compound, forms in starchy foods like potatoes, bread, cereals, crackers, and french fries when they are subjected to high-temperature cooking methods—including frying, baking, roasting, or grilling at temperatures exceeding 120 °C [1,2].

It has been established that ACR functions as a mutagen [3,4], carcinogen [5], genotoxin [6], neurotoxin [7], and reprotoxin [8], a combination of hazards that underlies its capacity for widespread organ damage. ACR also induces lung toxicity [9]. The lungs contribute to the metabolism of inhaled toxins by performing biotransformation and exhibiting high cytochrome P450 activity. Therefore, some of ACR’s toxic and mutagenic effects in pulmonary tissue may originate from this biotransformation process and the associated oxidative imbalance [10]. Inhalation of ACR can directly irritate and damage the respiratory system. Regardless of the exposure route, ACR exposure leads to oxidative stress and inflammation, largely due to the depletion of antioxidants such as glutathione (GSH), which can contribute to lung dysfunction and disease.

ACR exposure has been linked to lung dysfunction and elevated systemic inflammation in human populations [11]. While various antioxidants have been explored for their protective effects against ACR-induced toxicity, their clinical application may be constrained by limitations such as poor bioavailability, dose-dependent pro-oxidant effects, and insufficient lung targeting. N-acetylcysteine (NAC), a glutathione precursor with high pulmonary concentration and an established clinical safety profile, offers a promising therapeutic approach for ACR-induced lung injury.

N-Acetylcysteine (NAC) is a compound present at high concentration in lung tissue, where it acts as a direct precursor to the critical antioxidant glutathione (GSH) [12]. Beyond its role in GSH synthesis, NAC possesses intrinsic antioxidant and anti-inflammatory capabilities, as well as effective mucolytic properties [12]. A key pathological link in ALI is the depletion of GSH and the resulting oxidative stress, which correlates with adverse clinical outcomes [13]. NAC mitigates this damage by directly scavenging harmful ROS, leveraging its antioxidant and anti-inflammatory actions [14]. A critical but underexplored molecular hub in oxidative stress-induced lung injury is GSK-3β. GSK-3β serves as a crosstalk node between oxidative stress and inflammation by inhibiting the nuclear translocation and transcriptional activity of Nrf2 (the master regulator of antioxidant defense) while simultaneously facilitating NF-κB activation and subsequent pro-inflammatory cytokine production. This dual role positions GSK-3β as a relevant therapeutic target in ACR-induced pulmonary toxicity; however, no previous study has investigated whether NAC exerts its protective effects through modulation of the GSK-3β/Nrf2/NF-κB axis in the lung. Based on these considerations, the present study was designed to investigate the protective effects of NAC against acrylamide-induced lung toxicity in a rat model. Special emphasis was placed on elucidating the molecular mechanisms underlying NAC-mediated protection, particularly its regulatory role on the GSK-3β/Nrf2/NF-κB signaling pathway, which represents a critical axis linking oxidative stress and inflammation. The study further aimed to provide integrated molecular and immunohistochemical evidence supporting the ability of NAC to enhance antioxidant defense through activation of Nrf2 and its downstream targets (HO-1 and NQO1) while suppressing NF-κB-driven inflammatory responses, thereby attenuating ACR-induced pulmonary injury.

## 2. Materials and Methods

### 2.1. Animals

A total of forty adult male Wistar rats, with an initial body weight of 180 ± 20 g, were used in this study. The animals were randomly assigned to housing and provided with ad libitum access to standard rodent chow and water. Following a two-week acclimatization period, the rats were maintained under controlled environmental conditions (room temperature of 25 °C with a 12 h light/dark cycle) for the duration of the experiment. All experimental procedures were conducted in strict accordance with the ARRIVE guidelines, and in compliance with both the U.K. Animals (Scientific Procedures) Act, 1986 and the European Union Directive 2010/63/EU on the protection of animals used for scientific purposes. The study protocol received prior approval from the IACUC at Kafrelsheikh University, Egypt (Approval No. KFS-IACUC/341/2026).

### 2.2. Study Design

This study consisted of four groups, each containing ten rats (*n* = 10), and was conducted over an 11-day experimental period. Group I (Control) received 1 mL of 0.9% normal saline intragastrically once daily. Group II (NAC group) received NAC; Sigma Aldrich, St. Louis, MO, USA) dissolved in sterile 0.9% normal saline and administered orally at a dose of 250 mg/kg/day, following the protective protocol described by Altinoz et al. [15], with the total gavage volume standardized to 1 mL per rat. A single dose was selected based on this validated protocol to investigate molecular mechanisms rather than to establish a dose–response relationship. Group III (ACR group) received acrylamide (ACR; purchased from Sigma Chemical Co., St. Louis, MO, USA; purity ≥ 99%) dissolved in normal saline and administered intragastrically at a dose of 50 mg/kg for 11 consecutive days; this dosage was selected based on the established ACR-induced lung toxicity model reported by Atasever et al. [16]. This model is well-validated by multiple previous studies [9,16]; therefore, a separate positive control group was not required. Group IV (NAC + ACR group) received both NAC and ACR at the same doses, co-dissolved in the same 1 mL normal saline solution, and administered via the same intragastric route described for Groups II and III. At the end of the experimental period, all rats were euthanized by decapitation under light sevoflurane anesthesia, and lung tissues were collected. For histopathological evaluation, lung samples were washed with physiological saline, fixed in 10% formaldehyde, processed, and embedded in paraffin blocks. For biochemical and molecular analyses, the lung tissues were washed with physiological saline and stored at −80 °C until further use.

### 2.3. Assessment of Oxidative Stress Biomarkers in Lung Tissue

To evaluate oxidative stress, lung tissue samples were first flash-frozen in liquid nitrogen and mechanically pulverized using a Tissue Lyser II (Qiagen, Venlo, The Netherlands). The pulverized tissue was homogenized in a chilled buffer solution. Homogenates were subjected to differential centrifugation to prepare supernatants for specific assays: for MDA, SOD, and CAT analyses, samples were centrifuged at 3500 rpm for 15 min at 4 °C; for GSH and GPx measurements, centrifugation was performed at 10,000 rpm for 20 min at 4 °C. The enzymatic activities of SOD, CAT, and GPx were determined spectrophotometrically according to established methods [17,18,19]. The concentrations of MDA and GSH were quantified using colorimetric assays as described by Placer et al. [20] and Sedlak and Lindsay [21], respectively. Total protein content in the supernatant was measured using the method of Lowry et al. [22] to normalize biochemical data. All measurements were conducted in duplicate.

### 2.4. Histopathological Study

At the conclusion of the study, lung tissue samples were preserved in a 10% formaldehyde solution for 48 h. Following standard histological protocols, the tissues were embedded in paraffin. Sections of 4 μm thickness were prepared from these blocks and stained with H&E for analysis using a light microscope. In addition to H&E staining, PAS staining was performed to assess goblet cell activity, mucopolysaccharide content, and basement membrane alterations, while Masson’s trichrome staining was used to evaluate collagen deposition and fibrotic changes within the lung parenchyma. The following H&E-stained sections parameters were evaluated: alveolar wall thickening/edema, leukocytic infiltration, vascular congestion, and epithelial hyperplasia. Each parameter was scored on a four-point scale (0 = absent, 1 = mild, 2 = moderate, 3 = severe) according to Schafer et al. [23], based on the percentage of field affected (<25%, 25–50%, >50%).

### 2.5. Immunohistochemical Examination

Immunohistochemical (IHC) analysis was performed to evaluate the expression of Caspase 3, NF-κB, Nrf2, and IL-1β in lung tissues, following a previously described protocol [24]. Paraffin-embedded lung Sections (5 μm thick) were deparaffinized and rehydrated through a graded ethanol series (100%, 95%, 70%), then rinsed in distilled water. The sections were washed with phosphate-buffered saline (PBS), and endogenous peroxidase activity was quenched using 0.1% H_2_O_2_ for 30 min at room temperature.

To reduce nonspecific background staining, sections were incubated with 10% normal goat serum for 1 h at room temperature. Primary antibodies were applied for 60 min at room temperature: Caspase 3 (Cat# GB11532, 1:500, ServiceBio Technology Co., Ltd., Wuhan, China), NF-κB (Cat# bs-0465R, 1:500, Bioss Antibodies, Woburn, MA, USA), Nrf2 (Cat# ab313825, 1:100, Abcam, Cambridge, UK), and IL-1β (Cat# orb382131, 1:200, Biorbyt, Cambridge, UK). All antibodies were selected based on manufacturer validation for rat tissue reactivity. After PBS washing, sections were incubated with the appropriate secondary antibodies for 20 min, followed by streptavidin–HRP conjugate for 10 min. Immunoreactivity was visualized using DAB as the chromogen, and sections were counterstained with hematoxylin. Negative control sections were prepared by replacing the primary antibody with PBS (antibody diluent), while all other IHC steps were performed identically. This confirmed the specificity of the immunostaining.

Digital images were captured by a blinded pathologist at the Department of Pathology, Faculty of Veterinary Medicine, Mansoura University, using an Olympus imaging system. Quantitative assessment of immunopositivity was performed by measuring the percentage of positive immunoexpression area using ImageJ software Fiji 2.0.1. To ensure unbiased field selection, five random microscopic fields per section were selected using a systematic random sampling method. Specifically, each lung section was systematically scanned from the upper-left corner to the lower-right corner, and fields were selected at regular intervals (every 3–5 fields) to ensure uniform representation of the entire tissue section. Fields containing major artifacts (e.g., tissue folds, air bubbles, or uneven staining) were excluded. Multiple microscopic fields from at least three animals per group were analyzed, and the mean percentage of immunopositive area was calculated for each animal in accordance with published quantification methods. All image analyses were performed by an investigator blinded to the experimental group assignments.

### 2.6. Analysis of Inflammation Markers

Concentrations of the inflammation-related markers TNF-α, IL-6, Bax, Bcl-2, and GSK3beta in lung tissue supernatants were determined using corresponding ELISA kits in accordance with the manufacturers’ guidelines. Absorbance was measured at 450 nm with a Bio-Tek ELISA plate reader (Winooski, VT, USA). The kits provided the following analytical ranges and sensitivities: TNF-α (Cat. No ER1393: 3.906–250 pg/mL, sensitivity 2.344 pg/mL), IL-6 (Cat. No ELR-IL6: 30–10,000 pg/mL, sensitivity 30 pg/mL), Bax (Cat. No LS-F5064-1: 0.312–20 ng/mL, sensitivity 0.312 ng/mL), Bcl-2 (Cat. No LS-F4135-1: 78.13–5000 pg/mL, sensitivity 28 pg/mL), and GSK3β (Cat. No MBS766198: 0.156–10 ng/mL, sensitivity 0.094 ng/mL).

### 2.7. Quantitative Real-Time PCR (qRT PCR)

Quantitative real time PCR (qRT PCR) analysis was performed according to our previously published protocol [25]. Total RNA was extracted from homogenized lung tissue samples of all groups using the QIAzol reagent (Qiagen, Hilden, Germany) according to the manufacturer’s protocol, and then RNA purity and concentration were determined using a NanoDrop (Thermo Fisher Scientific, Waltham, MA, USA) by measuring absorbance at 260 nm and the A260/280 ratio. Reverse transcription of 1 μg RNA into cDNA was performed according to the manufacturer’s instructions of the SensiFAST™ cDNA Synthesis Kit (Bioline, London, UK). Finally, cDNA templates were amplified using a real-time PCR apparatus (Applied Biosystems 7500, Foster City, CA, USA) with an amplification profile of the following: initial denaturation of 2 min at 95 °C followed by 40 cycles of 10 s at 95 °C and 30 s at 60 °C. The amplification reaction contained 10 μL of HERA SYBR green PCR Master Mix (Willowfort, Birmingham, UK), 2 μL of cDNA, 1 µL of forward primer, 1 µL of reverse primer and 6 μL of nuclease-free water in a total reaction volume of 20 µL. Specific primers were used for HO 1 (NM_012580.2; forward 5′ ACAGGGTGACAGAAGAGGCTAA 3′, reverse 5′ CTGTGAGGGACTCTGGTCTTTG 3′), NF-κB (NM_199267.2; forward 5′ TCCTGTTCGAGTCTCCATGCAG 3′, reverse 5′ GGTCTCATAGGTCCTTTTGCGC 3′), Nrf2 (NM_031789.3; forward 5′ CACATCCAGACAGACACCAGT 3′, reverse 5′ CTACAAATGGGAATGTCTCTGC 3′), NQO1 (NM_017000.3; forward 5′ ATTGTATTGGCCCACGCAGA 3′, reverse 5′ TCATATCCCAGGCCACCTGA 3′), and GAPDH as the housekeeping gene (NM_017008.4; forward 5′-CCGCATCTTCTTGTGCAGTG-3′, reverse 5′ GAGAAGGCAGCCCTGGTAAC 3′). Primers were designed using Primer3 software (v.4.1.0; http://primer3.ut.ee/). Primer blast software (https://www.ncbi.nlm.nih.gov/tools/primer-blast/ (accessed on 27 May 2026)) was used for checking the primer specificity, and melting curve analysis was done. The relative quantitation (RQ) of mRNA expression of HO 1, NF-κB, Nrf2, and NQO1 genes was calculated using the 2^−∆∆Ct^ method [26]. All gene expression values were standardized using GAPDH as the housekeeping reference gene.

### 2.8. Statistical Analysis

Statistical analyses were conducted using GraphPad Prism software (version 8.0). Data are presented as mean ± SD. Normality of data distribution was assessed using the Shapiro–Wilk test. Intergroup comparisons were performed using one-way ANOVA, followed by Tukey’s post hoc test for multiple comparisons. A probability (*p*) value of less than 0.05 was considered statistically significant.

## 3. Results

### 3.1. Ameliorative Effect of NAC on ACR-Induced Lung Injury and Fibrosis

**Figure 1:**
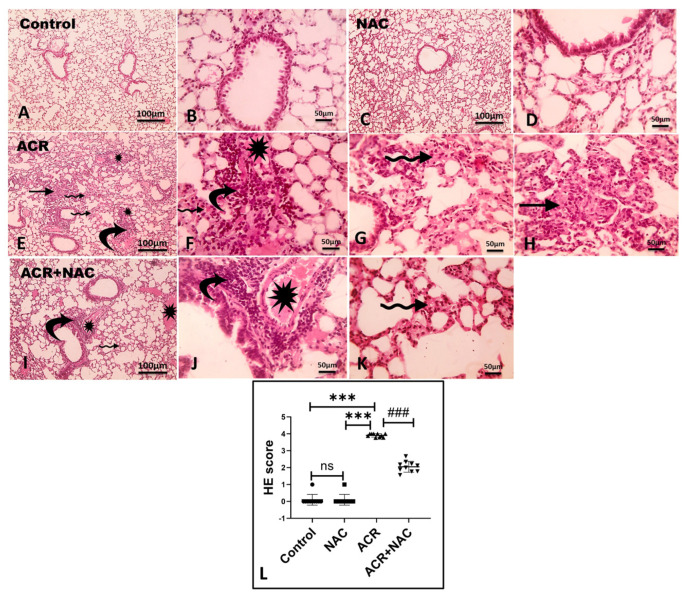
(**A**–**D**) Lung sections stained with H&E showing normal bronchioles, alveolar walls and alveolar lumina in control normal groups and NAC groups (n = 10). (**E**–**H**) Lung sections from the ACR group (n = 10) showing thickened edematous alveolar wall (zigzag arrow), congested blood vessels (Astrexs) with perivascular aggregation of leukocytic cells (curved arrows), focal area of hyperplastic alveolar epithelium and septal cells (thin arrow). (**I**–**K**) Lung sections from ACR + NAC group (n = 10) showing congested blood vessels (asterixs) with decreased perivascular aggregation of leukocytic cells (curved arrows). (**L**) Lung score. Magnifications: ×: 100 bar 100 and ×: 400 bar 50. *** denotes a very strong statistical difference relative to the control and NAC groups. ### denotes a very strong statistical difference relative to ACR-treated group.

**Figure 2:**
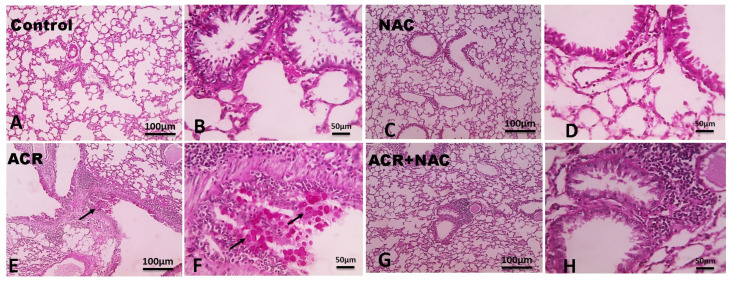
(**A**–**D**) Lung sections stained with PAS showing normal bronchiolar epithelium in control normal group and NAC groups (n = 10). (**E**,**F**) Lung sections from ACR group (n = 10) showing increased goblet cells in bronchiolar epithelium (thin arrow). (**G**,**H**) Lung sections from ACR + NAC group (n = 10) showing normal bronchiolar epithelium. Magnifications: ×: 100 bar 100 and ×: 400 bar 50.

**Figure 3:**
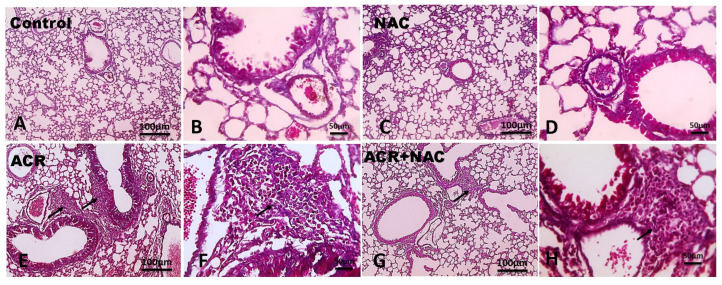
(**A**–**D**) Lung sections stained with MT showing normal bronchioles with no fibrosis in control normal group and NAC groups (n = 10). (**E**,**F**) Lung sections from ACR group (n = 10) showing increased peribrochiolar bluish collagen deposition (thin arrow). (**G**,**H**) Lung sections from ACR + NAC group (n = 10) showing markedly decreased peribrochiolar bluish collagen deposition (thin arrow). Magnifications: ×: 100 bar 100 and ×: 400 bar 50.

Histopathological examination of lung sections stained with H&E (Figure 1A–D) revealed normal lung architecture in both the control and NAC-treated groups, characterized by intact alveolar and bronchial walls and well-preserved alveolar lumina. PAS staining (Figure 2A–D) demonstrated normal epithelial features, while Masson’s trichrome staining confirmed the absence of fibrotic changes (Figure 3A–D). In contrast, the ACR-treated group exhibited pronounced histopathological alterations indicative of lung injury. These included alveolar wall edema, hyperplasia of alveolar epithelial and septal cells, leukocytic infiltration, and vascular congestion (Figure 1E–H). PAS staining further showed an increased number of goblet cells (Figure 2E,F), and Masson’s trichrome staining demonstrated evident peribronchiolar collagen deposition, appearing as bluish fibrotic areas (Figure 3E,F). Notably, the ACR + NAC co-treated group showed significant improvement. There was a highly significant reduction (*p* < 0.0001) in the histological degeneration score (Figure 1L) compared with the ACR group. Histologically, this improvement was reflected by marked reductions in vascular congestion and leukocytic infiltration (Figure 1I–K), restoration of normal bronchial epithelial features on PAS staining (Figure 2G,H), and diminished peribronchiolar collagen deposition on Masson’s trichrome staining (Figure 3G,H). Collectively, these findings demonstrate that NAC exerts a protective and restorative effect against ACR-induced lung injury and fibrosis.

### 3.2. Antioxidant Effect of NAC Against ACR-Induced Pulmonary Oxidative Stress

**Figure 4:**
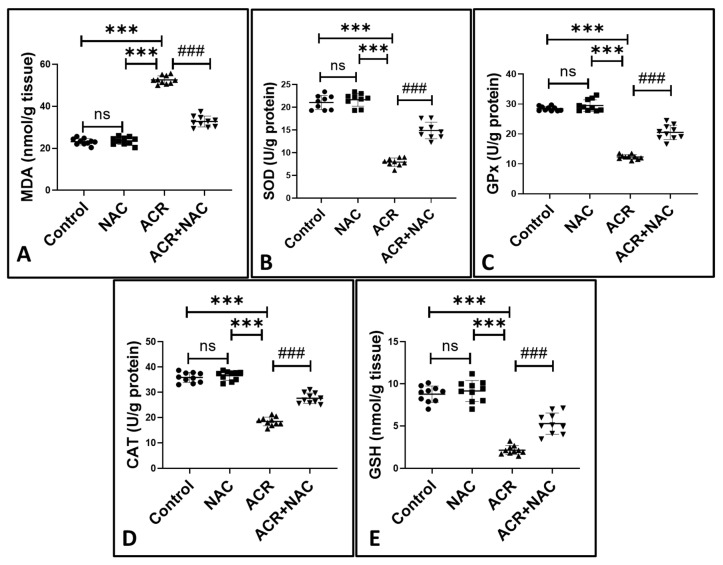
Impact of NAC and ACR on oxidative stress markers (**A**) MDA, (**B**) SOD, (**C**) GPx, (**D**) CAT, (**E**) GSH in lung tissues of different groups. Data are presented as mean ± SD (n = 10 rats per group). All data of different groups were analyzed by one way ANOVA followed by Tukey’s test to compare all means. *** denotes a highly significant difference relative to both the control group and the NAC group (*p* < 0.0001). ### denotes a highly significant difference relative to the ACR group (*p* < 0.0001).

Consistent with the findings shown in Figure 4, the ACR-treated group exhibited pronounced pulmonary oxidative stress, demonstrated by a highly significant elevation (*p* < 0.0001) in the lipid peroxidation marker MDA, along with a marked reduction in the antioxidant parameters SOD, GPx, CAT, and GSH compared with normal control animals. In contrast, the ACR + NAC group displayed a clear antioxidant response, reflected by a significant reduction in MDA levels and restoration of antioxidant enzyme activities.

**Figure 5:**
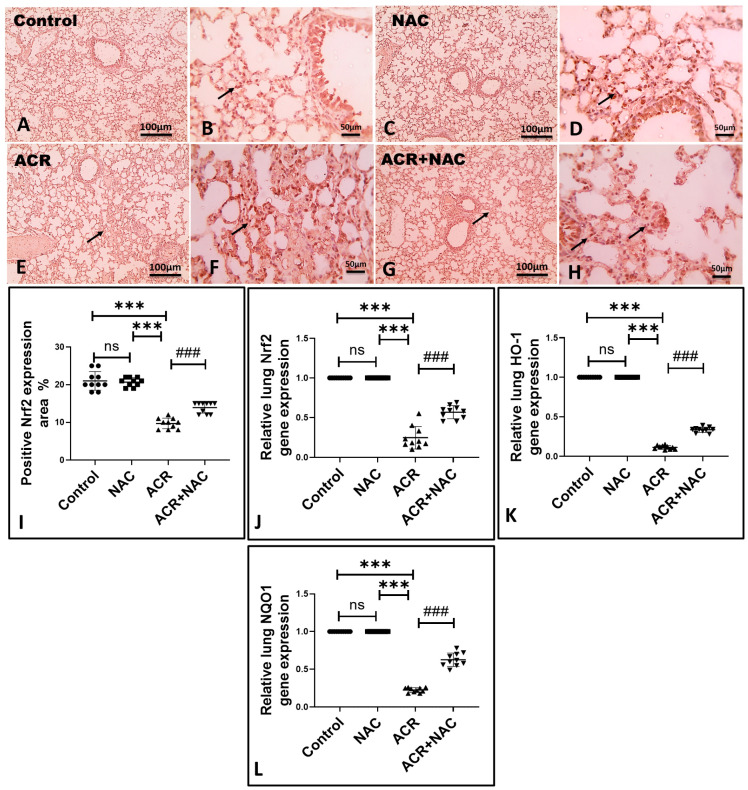
(**A**–**D**) Lung sections immunostained with Nrf2 showing normal positive brown expression (thin arrow) in alveolar wall in control normal group and NAC groups. (**E**,**F**) Lung sections from ACR group showing decreased positive brown expression for Nrf2 (thin arrow) in alveolar wall. (**G**,**H**) Lung sections from ACR + NAC group showing increased positive brown expression for Nrf2 in alveolar wall (thin arrow). IHC counterstained with Mayer’s hematoxylin. Magnifications: ×: 100 bar 100 and ×: 400 bar 50. (**I**) Bars represent statistical analysis of positive expression area % of Nrf2 in immunostained lung sections from different groups analyzed by one way ANOVA followed by Tukey’s test to compare all means. (**J**–**L**) Relative gene expression of Nrf2, HO-1, NQO1. Values are expressed as mean ± SD (n = 10). *** indicates that the values differ very significantly from the control and NAC groups (*p* < 0.0001). ### indicates a very significant difference when compared with the ACR-treated group (*p* < 0.0001).

The antioxidant mediated protective effect of NAC against ACR toxicity was further supported by the immunohistochemical and molecular analyses shown in Figure 5. Lung sections from control and NAC-treated groups exhibited normal positive brown immunoexpression of Nrf2 within the alveolar walls. Conversely, ACR-treated rats showed a noticeable decrease in Nrf2 immunoexpression. Notably, co-treatment with NAC restored and enhanced Nrf2 positivity in lung tissue. These findings were corroborated by the gene expression results, which demonstrated significant upregulation of Nrf2 and its downstream ARE HO 1 and NQO1 in the ACR + NAC group relative to the ACR group.

Altogether, these outcomes confirm that NAC exerts its protective antioxidant effect through activation of the Nrf2/HO 1/NQO1 signaling pathway, leading to enhanced transcription of endogenous antioxidant enzymes and subsequent elevation of antioxidant defenses in lung tissues.

### 3.3. Anti-Inflammatory Impact of NAC on ACR-Induced Lung Inflammation

**Figure 6:**
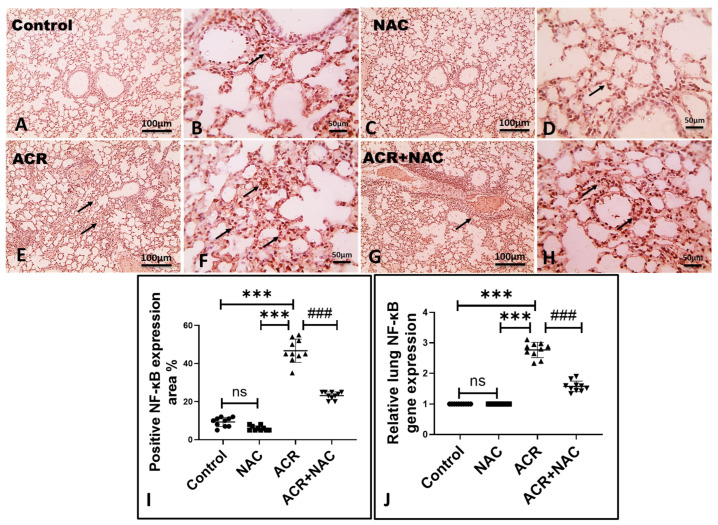
(**A**–**D**) Lung sections immunostained with NF-κB showing normal positive brown expression (thin arrow) in alveolar wall in control normal group and NAC group. (**E**,**F**) Lung sections from ACR group showing markedly increased positive brown expression for NF-κB (thin arrow) in alveolar wall. Lung sections from (**G**,**H**) ACR + NAC group showing decreased positive brown expression for NF-κB (thin arrow) in alveolar wall. IHC counterstained with Mayer’s hematoxylin. Magnifications: ×: 100 bar 100 and ×: 400 bar 50. (**I**) Bars represent statistical analysis of positive expression area %of NF-κB in immunostained lung sections from different groups analyzed by one way ANOVA followed by Tukey’s test to compare all means. (**J**) Relative gene expression of NF-κB. Results are presented as mean ± SD (n = 10). *** indicates a markedly significant difference compared with both the control and NAC groups. ### indicates a markedly significant difference compared with the ACR-treated group.

**Figure 7:**
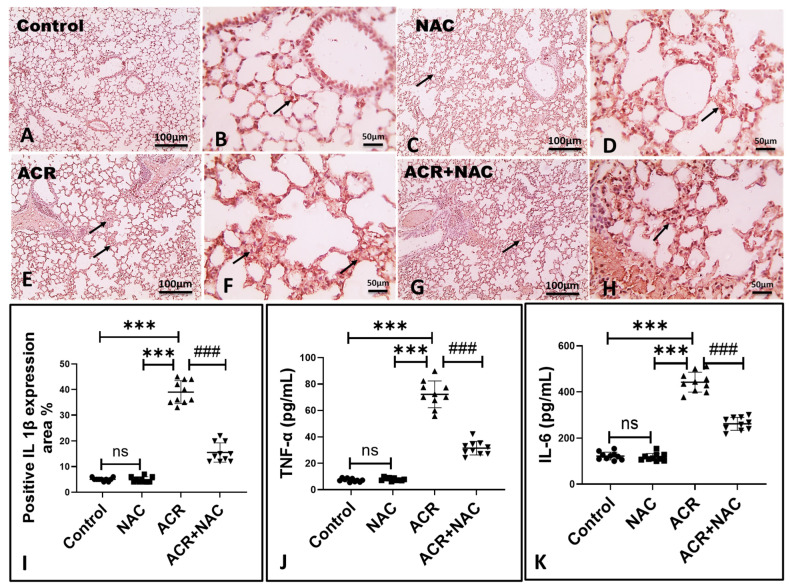
(**A**–**D**) Lung sections immunostained with IL-1β showing normal positive brown expression (thin arrow) in alveolar wall in control normal group and NAC groups (n = 10). (**E**,**F**) Lung sections from ACR group (n = 10) showing markedly increased positive brown expression IL-1b (thin arrow) in alveolar wall. (**G**,**H**) Lung sections from ACR + NAC group (n = 10) showing decreased positive brown expression IL-1β (thin arrow) in alveolar wall. IHC counterstained with Mayer’s hematoxylin. Magnifications: ×: 100 bar 100 and ×: 400 bar 50. (**I**) Bars represent statistical analysis of positive expression area % of IL-1β in immunostained lung sections from different groups analyzed by one way ANOVA followed by Tukey’s test to compare all means. (**J**,**K**) ELISA level of TNF-α, IL-6 in lung tissues of all groups. *** denotes a very strong statistical difference relative to the control and NAC groups. ### denotes a very strong statistical difference relative to ACR-treated group.

As shown in Figure 6, both the immunohistochemical expression and mRNA levels of NF-κB were markedly elevated in the ACR-treated group (*p* < 0.0001) compared with the control rats. This activation of NF-κB was accompanied by a significant increase in the immunoexpression of IL-1β and elevated protein levels of the pro-inflammatory cytokines TNF α and IL-6, as demonstrated in Figure 7. In contrast, the group that received the combined treatment of ACR + NAC showed a clear reduction in these inflammatory mediators. NAC co-administration significantly lowered the levels of NF-κB, TNF α, IL-1β, and IL-6, whether assessed through gene expression, immunoexpression, or ELISA measurements, compared with the ACR-only group. These findings support the conclusion that NAC exerts a potent anti-inflammatory effect, effectively mitigating ACR-induced pulmonary inflammation.

### 3.4. Antiapopotic Effect of NAC Against ACR-Induced Pulmonary Apoptosis

**Figure 8:**
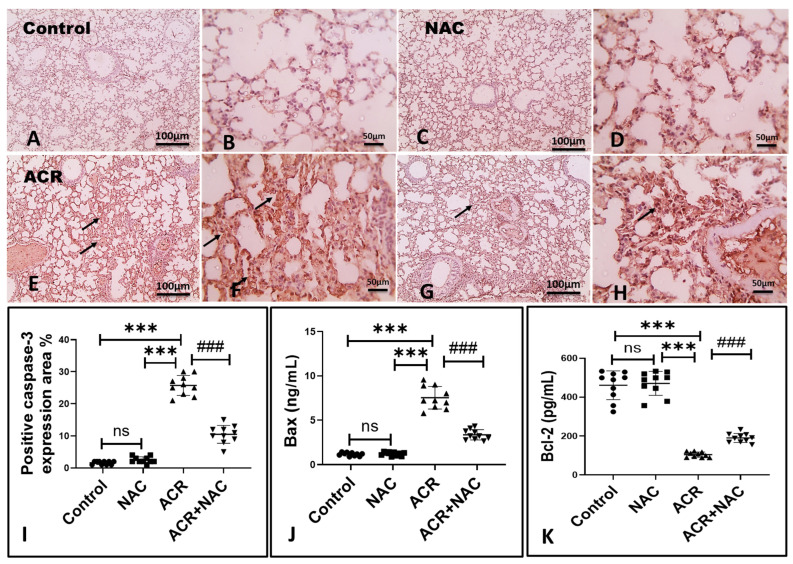
(**A**–**D**) Lung sections immunostained with caspase-3 showing negative expression in alveolar wall in control normal group and NAC groups (n = 10). (**E**,**F**) Lung sections from ACR group (n = 10) showing markedly increased positive brown expression for caspase-3 (thin arrow) in alveolar wall. (**G**,**H**) Lung sections from ACR + NAC group (n = 10) showing decreased positive brown expression for caspase-3 (thin arrow) in alveolar wall. IHC counterstained with Mayer’s hematoxylin. Magnifications: ×: 100 bar 100 and ×: 400 bar 50. (**I**) Bars represent statistical analysis of positive expression area % of caspase-3 in immunostained lung sections from different groups analyzed by one way ANOVA followed by Tukey’s test to compare all means. (**J**,**K**) ELISA level of Bax and Bcl-2. *** denotes a very strong statistical difference relative to the control and NAC groups. ### denotes a very strong statistical difference relative to ACR-treated group.

As shown in Figure 8, the ACR-treated group exhibited marked pulmonary apoptosis, evidenced by a significant (*p* < 0.0001) increase in caspase 3 immunoexpression and elevated protein levels of the proapoptotic marker Bax, along with a concomitant reduction in the antiapoptotic protein Bcl 2, as determined by ELISA, compared with normal control animals. Conversely, the ACR + NAC group demonstrated a clear anti-apoptotic effect of NAC, as indicated by the reversal of these apoptotic markers relative to the ACR group. This protective, antiapoptotic action of NAC in the ACR-induced pulmonary toxicity model is likely attributable to its potent antioxidant and anti-inflammatory properties.

### 3.5. Impact of ACR and NAC on GSK 3β Signaling

**Figure 9:**
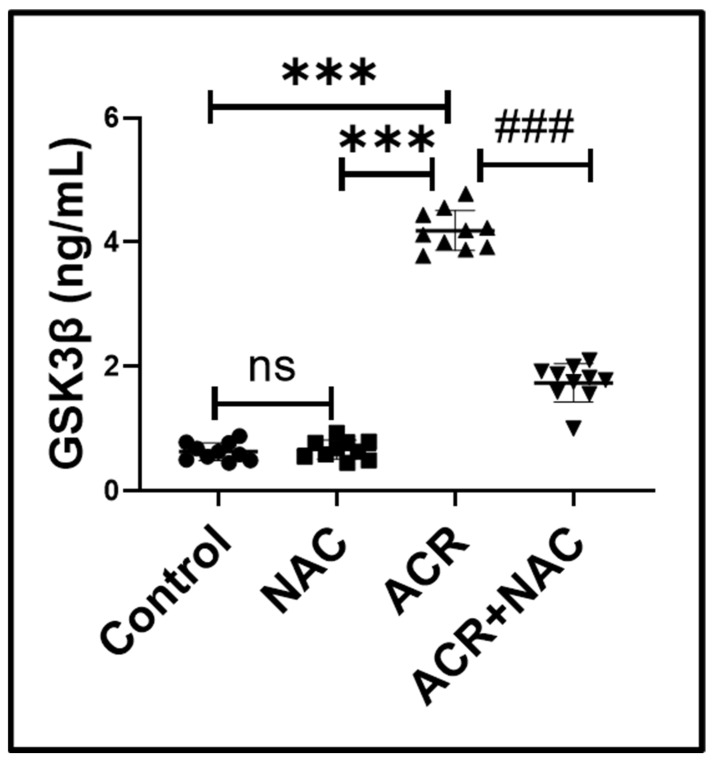
ELISA assay of GSK 3β in different groups. All data are expressed as mean ± SD (n = 10). *** strong statistical difference to normal groups. ### denotes a very strong statistical difference relative to ACR-treated group.

As presented in Figure 9, intragastric administration of ACR at a dose of 50 mg/kg resulted in a significant (*p* < 0.0001) upregulation of the GSK 3β signaling pathway compared with the control group. In contrast, animals receiving the combined treatment (ACR + NAC) showed a marked reduction in GSK 3β protein levels, as measured by ELISA, relative to the ACR-only group. This finding indicates that NAC effectively counteracts the ACR-induced upregulation of GSK 3β.

## 4. Discussion

Acrylamide (ACR) is a water-soluble and widely distributed toxic compound found in various foods [27]. Studies in animal models demonstrate that ACR can induce DNA damage and exert toxic effects on the nervous system, liver, kidneys, and testes [28,29,30,31]. In the lungs, ACR exposure has been shown to trigger cell death, inflammation, DNA damage, and alterations in the alveolar epithelium [32].

Oxidative stress plays a critical role in the development of numerous diseases via redox imbalance and subsequent cellular damage [33]. During the metabolic processing of ACR, ROS are generated, which attack polyunsaturated fatty acids in cellular membranes and initiate lipid peroxidation. One of the major end products of this process is MDA, a widely recognized marker of oxidative damage [34]. Previous studies have confirmed that ACR induces lipid peroxidation in red blood cells, leading to a measurable increase in MDA levels [35]. These findings are consistent with our results; intragastric administration of ACR at a dose of 50 mg/kg for eleven days was sufficient to induce oxidative degeneration in lung tissue, evidenced by elevated MDA levels and reduced antioxidant parameters.

Conversely, co-administration of NAC with ACR markedly attenuated this oxidative injury. This outcome aligns with the results of Altinoz et al. [15], who demonstrated the antioxidant effect of NAC against ACR-induced hepatic and intestinal oxidative stress. The antioxidant efficacy of NAC is largely attributed to its ability to enhance intracellular GSH levels and stimulate the activity of the GST enzyme. This mechanism is further supported by findings from Eskiocak et al. [36], who showed that pharmacological doses of NAC significantly reduced lipid peroxidation while increasing GSH concentrations, a key determinant in mitigating oxidative stress.

The pulmonary toxicity caused by ACR is also closely linked to oxidative stress [37]. ACR and its metabolite, glycidamide, interact with and deplete cellular GSH stores, resulting in increased levels of MDA, a major indicator of oxidative injury in ACR-exposed rats [38]. In response to this oxidative challenge, activation of the Nrf2 signaling pathway serves as a crucial compensatory defense mechanism. Upon activation, Nrf2 detaches from its cytoplasmic inhibitor Keap1 translocates into the nucleus, and binds to the ARE, thereby stimulating the transcription of phase II detoxifying and antioxidant enzymes, including those involved in GSH synthesis and regeneration [39]. Key downstream targets of Nrf2 include heme oxygenase 1 (HO 1) and NAD(P) H: quinone oxidoreductase 1 (NQO 1), both of which play vital roles in modulating oxidative injury and cellular survival pathways [40].

Our findings support this mechanistic framework: the ACR-only group demonstrated a significant decrease in Nrf2 mRNA expression and immunoreactivity in lung tissue, accompanied by reduced gene expression of ARE-regulated targets (HO 1, NQO 1) compared with normal lung tissues. These observations are in agreement with Baraka et al. [41], who reported the detrimental effect of ACR on Nrf2/NQO1/HO 1 signaling in brain tissues. In contrast, the NAC + ACR group exhibited marked improvement in Nrf2 pathway activity, consistent with the study of Messier et al. [42], which highlighted the protective role of NAC in preserving type II pneumocytes in a murine model of cigarette smoke-induced lung injury. This mechanism is corroborated by evidence showing that NAC treatment increases Nrf2 expression while reducing KEAP1 levels under oxidative stress conditions, and that NAC derivatives such as NACA enhance Nrf2 nuclear activation following oxidative insults [43,44].

Based on the research provided, the transcription factor NF-κB is a central regulator of inflammation, responsible for activating the expression of several pro-inflammatory mediators, including COX 2, IL-1β, TNF α, IL-6, and iNOS [45,46,47]. Exposure to cytotoxic agents such as ACR has been shown to induce the upregulation of these inflammatory molecules, including NF-κB, IL-1β, TNF α, COX 2, and IL-6. Consistent with this, the present study demonstrates that oral administration of ACR for 11 days markedly induces lung inflammation, as evidenced by elevated NF-κB gene expression and immunostaining, alongside a significant increase in pro-inflammatory cytokines (TNF α, IL-6, IL-1β). These findings align with the observations of Yesildag et al. [9].

In contrast, co-treatment with NAC exerted a potent anti-inflammatory effect, reversing all ACR-induced inflammatory markers, a finding supported by earlier studies [48]. The suppressive effect of NAC on NF-κB activation can be attributed to its antioxidant capacity; by lowering intracellular ROS levels, NAC inhibits the redox-sensitive signaling events required for NF-κB activation [49]. Additionally, NAC reduces TNF α expression, and because TNF α is one of the strongest upstream activators of NF-κB, this reduction indirectly contributes to further suppression of NF-κB signaling [50].

Apoptosis, or programmed cell death, is triggered by both intrinsic and extrinsic signals. A key mediator of this process is caspase 3 (CASP3), a central executioner protease responsible for orchestrating the downstream events of apoptosis [34]. Activation of caspase 3 is tightly regulated by shifts in pro- and anti-apoptotic molecules, including an increase in cytochrome c release, Bax expression, together with a reduction in the anti-apoptotic protein Bcl-2 [7]. In toxicological models, exposure to ACR significantly elevates cytoplasmic caspase 3 levels, indicating marked activation of the apoptotic cascade [51]. Our findings are consistent with these observations, as ACR administration induced pronounced lung apoptosis.

However, NAC demonstrated a protective, anti-apoptotic effect against ACR-induced lung injury in our study. This restorative effect is supported by previous research, including the work of Le et al. [52] and Chiang et al. [53], both of which reported that NAC attenuates caspase 3 activation and modulates apoptotic signaling pathways. Together, these findings reinforce the role of NAC as an effective inhibitor of apoptosis through its antioxidant and signaling modulatory properties.

Acrylamide exposure is known to induce oxidative stress, a condition that activates several redox sensitive kinases, including glycogen synthase kinase 3β (GSK 3β). Although direct ACR-specific investigations on GSK 3β are limited, substantial evidence demonstrates that GSK 3β is a key pro-inflammatory kinase whose upregulation intensifies inflammatory signaling. It is strongly associated with promoting a pro-inflammatory cellular phenotype and acts synergistically with NF-κB to amplify inflammatory responses [54,55]. Mechanistically, elevated GSK 3β activity enhances NF-κB activation by facilitating NF-κB nuclear accumulation and increasing transcription of downstream pro-inflammatory cytokines.

In addition to promoting NF-κB activation, GSK 3β functions as a suppressor of Nrf2, the master regulator of antioxidant defense. It phosphorylates Nrf2, targeting it for degradation and thereby weakening the antioxidant response. This mechanism is supported by studies showing that inhibition of GSK 3β enhances Nrf2 signaling, whereas active GSK 3β diminishes Nrf2-dependent antioxidant enzyme expression, including HO 1 and NQO 1 [56]. These findings align with our results, in which ACR exposure increased NF-κB while reducing Nrf2/HO 1 expression in lung tissues, consistent with the observations of Yan et al. [57]. The GSK-3β/Nrf2/NF-κB axis has been implicated in other lung injury models beyond ACR exposure. In LPS-induced ALI, pharmacological inhibition of GSK-3β has been shown to restore Nrf2 nuclear translocation and suppress NF-κB-dependent cytokine production [56].

Meanwhile, co-administration of NAC with ACR resulted in a significant decrease in GSK 3β protein levels in lung tissue. This decrease may help explain NAC’s antioxidant effect through upregulation of Nrf2, and its anti-inflammatory activity through downregulation of NF-κB, in addition to its well-established mechanisms of action. The inhibitory effect of NAC on GSK 3β can be attributed to its strong antioxidant capacity, which reduces oxidative stress and thereby prevents activation of redox-sensitive kinases such as GSK 3β. We acknowledge that only a single dose of NAC (250 mg/kg/day) was tested in this study. This dose was selected based on the established protective protocol of Altinoz et al. [15] to focus on mechanistic pathway elucidation. However, the protective effects of NAC are likely dose-dependent. Previous studies have reported that low-dose NAC protects against oxidative stress, whereas very high doses may paradoxically exhibit pro-oxidant effects or increase mortality in certain experimental models [36,37]. The dose used in our study (250 mg/kg/day) falls within the range reported to be safe and effective. Future studies are warranted to investigate the dose–response relationship of NAC against ACR-induced lung injury and to determine the optimal therapeutic dose. Additional limitations of this study include the lack of in vitro experiments using pulmonary epithelial cell lines (e.g., A549 or BEAS-2B), which would allow for direct mechanistic interrogation of the GSK-3β/Nrf2/NF-κB axis under controlled conditions. Furthermore, we did not investigate upstream regulators of GSK-3β, such as PI3K/Akt (which phosphorylates and inhibits GSK-3β at Ser9), nor downstream effectors beyond Nrf2 and NF-κB, including β-catenin signaling. Regarding GSK-3β pathway confirmation, we measured only total GSK-3β by ELISA. Western blot is the field standard for this type of mechanistic analysis. Due to lack of access to Western blot analysis, confirmatory experiments were not possible at this time. We acknowledge this limitation and will address it in future work. Future studies should employ in vitro models with targeted manipulation of GSK-3β (e.g., pharmacological inhibitors such as TDZD-8 or SB216763, siRNA knockdown, or overexpression) to establish causality, and should explore whether NAC activates the PI3K/Akt/GSK-3β axis, leading to β-catenin stabilization.

In humans, NAC has an established safety profile. It is FDA-approved for the treatment of acetaminophen overdose and as a mucolytic agent. Oral NAC is generally well-tolerated, with mild gastrointestinal side effects (nausea, vomiting, diarrhea) being the most common. Rare adverse effects include bronchospasm (particularly with nebulized administration), headache, and rash. Clinically relevant drug interactions include a potential reduction in the efficacy of nitroglycerin due to vasodilatory antagonism. The dose used in this study (250 mg/kg/day in rats) translates to approximately 40 mg/kg/day in humans using allometric scaling, which aligns with the range of clinically used doses (600–1800 mg/day). However, the present study used an acute ACR toxicity model that does not directly reflect chronic dietary exposure. Therefore, caution should be exercised when extrapolating these findings to human dietary ACR exposure. Furthermore, the ACR dose used in this study (50 mg/kg/day for 11 days) represents acute toxicity rather than chronic dietary exposure levels, which further limits the translational implications of our findings. These safety data support the translational potential of NAC for preventing chemically induced lung injury, although further human studies are warranted.

Our findings are consistent with and extend previous studies on NAC and ACR toxicity. Several earlier investigations have examined the protective effects of various antioxidants against ACR-induced organ toxicity. For example, a study on experimental tissue injury models demonstrated that oxidative stress and inflammation are key mediators of chemically induced damage, and that NAC effectively mitigates these effects [58]. Additionally, the role of Nrf2 activation in counteracting ACR toxicity has been highlighted in hepatic and neural tissues [41,59]. The present study is the first to specifically evaluate the GSK-3β/Nrf2/NF-κB axis in ACR-induced lung injury and its modulation by NAC, thereby providing a novel mechanistic insight.

## 5. Conclusions

**Figure 10:**
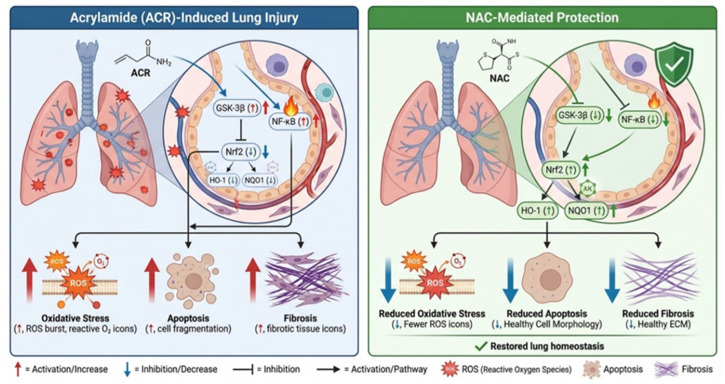
Acrylamide (ACR) triggers lung injury by activating GSK-3β, suppressing the Nrf2 antioxidant pathway, and promoting NF-κB inflammation, leading to oxidative stress, apoptosis, and fibrosis. N-Acetylcysteine (NAC) restores redox balance by downregulating GSK-3β, reactivating Nrf2/HO-1/NQO1 signaling, and suppressing NF-κB, thereby protecting the lung from chemical toxicity.

This study demonstrates that ACR induces marked lung injury characterized by oxidative stress, inflammation, apoptosis, fibrosis, and activation of GSK 3β signaling. NAC effectively mitigated these pathological alterations by restoring lung architecture, reducing collagen deposition, and normalizing epithelial features. It also exerted potent antioxidant, anti-inflammatory, and anti-apoptotic effects through activation of the Nrf2/HO 1/NQO1 pathway and suppression of NF-κB signaling. Additionally, NAC significantly downregulated ACR-induced GSK 3β activation, further contributing to its protective effects. Overall, NAC provides strong protective and therapeutic potential against ACR-induced pulmonary toxicity (Figure 10).

## Data Availability

The data supporting the findings of this study are available from the corresponding author upon reasonable request.

## Abbreviations

**Abbreviation**	**Full Form**	**Abbreviation**	**Full Form**
**ACR**	Acrylamide	**HO-1**	Heme Oxygenase-1
**ALI**	Acute Lung Injury	**HRP**	Horseradish Peroxidase
**ANOVA**	Analysis of Variance	**H_2_O_2_**	Hydrogen Peroxide
**ARE**	Antioxidant Response Element	**IACUC**	Institutional Animal Care and Use Committee
**ARRIVE**	Animal Research: Reporting of In Vivo Experiments	**IHC**	Immunohistochemistry
**Bax**	Bcl-2-associated X protein	**IL-1β**	Interleukin-1 beta
**Bcl-2**	B-cell lymphoma 2	**IL-6**	Interleukin-6
**CAT**	Catalase	**iNOS**	Inducible Nitric Oxide Synthase
**cDNA**	Complementary DNA	**KEAP1**	Kelch-like ECH-associated protein 1
**COX-2**	Cyclooxygenase-2	**LPS**	Lipopolysaccharide
**DAB**	3,3′ Diaminobenzidine	**MDA**	Malondialdehyde
**ELISA**	Enzyme-Linked Immunosorbent Assay	**mRNA**	Messenger Ribonucleic Acid
**FDA**	Food and Drug Administration (US)	**MT**	Masson’s Trichrome
**GAPDH**	Glyceraldehyde-3-Phosphate Dehydrogenase	**NAC**	N-Acetylcysteine
**GPx**	Glutathione Peroxidase	**NACA**	N-Acetylcysteine Amide
**GSH**	Reduced Glutathione	**NF-κB**	Nuclear Factor kappa-light-chain-enhancer of activated B cells
**GSK-3β**	Glycogen Synthase Kinase-3 beta	**NQO1**	NAD(P)H: Quinone Oxidoreductase 1
**GST**	Glutathione S-Transferase	**Nrf2**	Nuclear factor erythroid 2-related factor 2
**H&E**	Hematoxylin and Eosin	**PAS**	Periodic Acid–Schiff
**SOD**	Superoxide Dismutase	**PBS**	Phosphate-Buffered Saline
**TNF-α**	Tumor Necrosis Factor-alpha	**qRT-PCR**	Quantitative Real-Time Polymerase Chain Reaction
**SD**	Standard Deviation	**ROS**	Reactive Oxygen Species
**RQ**	Relative Quantitation		
